# Pulmonary PET imaging confirms preferential lung target occupancy of an inhaled bronchodilator

**DOI:** 10.1186/s13550-019-0479-8

**Published:** 2019-01-29

**Authors:** Magnus Schou, Pär Ewing, Zsolt Cselenyi, Markus Fridén, Akihiro Takano, Christer Halldin, Lars Farde

**Affiliations:** 1 PET Science Centre, Precision Medicine and Genomics, IMED Biotech Unit, AstraZeneca, Karolinska Institutet, Stockholm, Sweden; 2 Department of Clinical Neuroscience, Center for Psychiatry Research, Karolinska Institutet and Stockholm County Council, SE-171 76 Stockholm, Sweden; 3 Respiratory, Inflammation and Autoimmunity IMED Biotech Unit, AstraZeneca, Gothenburg, Sweden; 4 Translational PKPD, Department of Pharmaceutical Biosciences, Uppsala University, Uppsala, Sweden

**Keywords:** PET, Lungs, Muscarinic receptors, Ipratropium

## Abstract

**Background:**

Positron emission tomography (PET) is a non-invasive molecular imaging technique that traces the distribution of radiolabeled molecules in experimental animals and human subjects. We hypothesized that PET could be used to visualize the binding of the bronchodilator drug ipratropium to muscarinic receptors (MR) in the lungs of living non-human primates (NHP). The objectives of this study were two-fold: (i) to develop a methodology for quantitative imaging of muscarinic receptors in NHP lung and (ii) to estimate and compare ipratropium-induced MR occupancy following drug administration via intravenous injection and inhalation, respectively.

**Methods:**

A series of PET measurements (*n* = 18) was performed after intravenous injection of the selective muscarinic radioligand ^11^C-VC-002 in NHP (*n* = 5). The lungs and pituitary gland (both rich in MR) were kept in the field of view. Each PET measurement was followed by a PET measurement preceded by treatment with ipratropium (intravenous or inhaled).

**Results:**

Radioligand binding was quantified using the Logan graphical analysis method providing the total volume of distribution (*V*_*T*_). Ipratropium reduced the *V*_*T*_ in the lung and pituitary in a dose-dependent fashion. At similar plasma ipratropium concentrations, administration by inhalation produced larger reductions in *V*_*T*_ for the lungs. The plasma-derived apparent affinity for ipratropium binding in the lung was one order of magnitude higher after inhalation (*K*_*i*ih_ = 1.01 nM) than after intravenous infusion (*K*_*i*iv_ = 10.84 nM).

**Conclusion:**

Quantitative muscarinic receptor occupancy imaging by PET articulates and quantifies the therapeutic advantage of the inhaled route of delivery and provides a tool for future developments of improved inhaled drugs.

**Electronic supplementary material:**

The online version of this article (10.1186/s13550-019-0479-8) contains supplementary material, which is available to authorized users.

## Background

Chronic obstructive pulmonary disease is a progressive lung disorder characterized by breathing difficulties. It has been estimated that the disorder affects more than 300 million people worldwide [1], a number expected to increase over the coming years because of an aging population and increased smoking. There is currently no cure for chronic obstructive pulmonary disease, but its symptoms can be managed by inhalation of bronchodilating drugs, most commonly muscarinic antagonists (for long-term symptom management) and/or beta-adrenergic agonists (also used for quick symptom relief and as rescue medication). Despite their demonstrated clinical efficacy, the extent and duration of pulmonary receptor binding of bronchodilating drugs are poorly understood [2]. A major hurdle for understanding this in more detail is that plasma drug concentrations are unlikely to represent target site exposure and receptor binding in the lung tissue.

The non-invasive molecular imaging technique positron emission tomography (PET) is increasingly used in translational biomedical research and for clinical diagnoses. PET has so far had a limited impact on respiratory drug development, with exceptions for studies of lung deposition following inhalation of radiolabeled drugs or particles [3, 4]. A key strength with PET is that it permits quantification of drug binding to their respective target proteins in vivo. This exclusive information can in turn provide a means to derive relationships between drug concentrations in plasma, the degree of drug-induced target occupancy and optimal clinical effect. Though target occupancy studies are established in the development of drugs for the central nervous system [5], no such study has been reported for respiratory drug development.

The aim of the present study was to test the concept of estimating drug-induced target occupancy of a bronchodilator in the lung tissue and to provide a direct quantification of the preferential lung selectivity that results from the inhaled route of delivery when compared to systemic administration. For that purpose, we selected the reference drug ipratropium, an antagonist at muscarinic acetylcholine receptors, and studied its binding to lungs and the pituitary gland in non-human primates. To estimate ipratropium-induced muscarinic receptor occupancy, we used the previously developed radioligand, ^11^C-VC-002, that had been successfully applied in the study of muscarinic receptors in the human airways [6, 7]. To the best of our knowledge, this represents the first study that addresses the relationships between the route of administration, plasma drug concentration, and drug-induced receptor occupancy in vivo in pulmonary tissue.

## Materials and methods

### Animals and study design

Five female cynomolgus monkeys (mean body weight ± SD, 5.4 ± 0.9 kg, age 8.0 ± 1.4 years old) were examined in this study. The monkeys were owned by the Centre for Psychiatry Research, Department of Clinical Neuroscience, Karolinska Institutet, and housed in the Astrid Fagraeus Laboratory of the Swedish Institute for Infectious Disease Control, Solna, Sweden.

The study was divided into two panels, an intravenous (*n* = 4) and an inhalation panel (*n* = 5) (Tables 1 and 2). In the first panel, which comprised eight PET measurements performed in four sessions, radioligand binding in the lung was studied at baseline and following drug delivery via intravenous infusion. The drug, ipratropium (2–30 μg/kg), was infused intravenously over 15 min with start at 20 min before radioligand injection. In the second panel, which comprised ten PET measurements in five sessions, radioligand binding in the lung was studied at baseline and following drug delivery via inhalation. In this panel, ipratropium (0.5–150 μg/kg) was administered via a 15-min nebulization starting 20 min before radioligand injection. Each post-dosing PET measurement was performed 2.5 h after the baseline PET measurement.

**Table 1:** PET measurements in panel 1. Drug delivery via intravenous infusion

PET	NHP ID	NHP number	Post-dosing drug and dose
1	0407342	#1	N/A (baseline)
2	0407342	#1	Ipratropium (30 μg/kg)
3	0410288	#2	N/A (baseline)
4	0410288	#2	Ipratropium (2 μg/kg)
5	0606084	#4^*^	N/A (baseline)
6	0606084	#4	Ipratropium (2 μg/kg)
7	0412444	#3^*^	N/A (baseline)
8	0412444	#3	Ipratropium (10 μg/kg)

^*^Note: The monkeys are numbered according to the order of enrolment in the study hence the apparent swap in numbers

**Table 2:** PET measurements in panel 2. Drug delivery via inhalation

PET	NHP ID	NHP Number	Post-dosing drug and dose
1	0412444	#3	N/A (baseline)
2	0412444	#3	Ipratropium (150 μg/kg)
3	0610010	#5	N/A (baseline)
4	0610010	#5	Ipratropium (10 μg/kg)
5	0407342	#1	N/A (baseline)
6	0407342	#1	Ipratropium (2 μg/kg)
7	0610010	#5	N/A (baseline)
8	0610010	#5	Ipratropium (0.5 μg/kg)
9	0407342	#1	N/A (baseline)
10	0407342	#1	Ipratropium (1.5 μg/kg)

### Radioligand synthesis

^11^C-VC-002 was prepared as previously described [8]. The radiochemical purity of ^11^C-VC-002 exceeded 99% at time of injection, and the total injected mass varied between 0.12 and 0.16 μg, *n* = 18.

### PET experimental procedure

Anesthesia was induced by intramuscular injection of ketamine hydrochloride (approximately 10 mg/kg) and maintained by the administration of a mixture of sevoflurane (2–8%), oxygen, and medical air after endotracheal intubation. The head was immobilized with a fixation device [9]. Body temperature was maintained by a Bair Hugger model 505 (Arizant Healthcare, MN) and monitored by an esophageal thermometer. Heart rate, respiratory rate, and oxygen saturation were continuously monitored throughout the experiments. Blood pressure was monitored every 15 min. Fluid balance was maintained by a continuous infusion of saline.

In each PET measurement, a sterile physiological phosphate-buffered (pH 7.4) saline (PBS) solution containing a radiolabeled drug, in a volume not exceeding 5 mL, was injected as a bolus into a sural vein during 5 s. PET data acquisition started at the time of the bolus injection.

### PET measurements

PET measurements were conducted using the high-resolution research tomograph (HRRT) (Siemens Molecular Imaging). List mode data were reconstructed using the ordinary Poisson-3D-ordered subset expectation maximization algorithm, with 10 iterations and 16 subsets including modeling of the point spread function. The corresponding in-plane resolution was 1.5-mm full width at half-maximum in the center of the field of view and 2.4 mm at 10-cm off-center directions [10, 11].

A transmission scan of 6 min using a single ^137^Cs source was performed immediately before radioligand injection. List mode data were acquired continuously for 63 min immediately after iv injection of the radioligand. Images were reconstructed with a series of frames (10 s × 9, 15 s × 2, 20 s × 3, 30 s × 4, 1 min × 4, 3 min × 4, and 6 min × 7).

Regions of interest (ROIs) were delineated for the lung and brain on the summation PET images. The ROIs were applied to the dynamic PET data, and the time-activity curves of each organ were generated.

### Drug administration via inhalation

An Aeroneb Pro™ nebulization device (Dolema AB, Sweden) was used for the inhalation experiments. In brief, the Aeroneb Pro™ was connected to the ventilation unit using a T-junction via a pediatric intubation tube with an internal diameter of 4 mm. A solution of ipratropium bromide (4 mL) was then nebulized into the endotracheal tube for 15 min.

### Arterial and venous blood sampling

Monkeys were cannulated in the femoral artery or an artery of the lower limb. To avoid compromising animal health and/or outcome parameters, the aspired blood volume did not exceed 0.7% of the body weight. Saline was used as a volume replacement for the aspired blood.

Arterial blood was collected continuously during the first 3 min of each PET measurement using an automated blood sampling system (ABSS) (Allogg, Mariefred, Sweden). The ABSS pump speed was set to 3 mL/min. Thereafter, arterial blood samples (1–3 mL) were obtained manually at 5, 10, 20, 40, and 60 min for the measurement of radioactivity in whole blood and plasma. Blood samples (1–2 mL) at 2.5, 10, 20, 40, and 60 min were also used for radioligand metabolite analysis. Ipratropium was quantitated in plasma using LC-MS/MS. Non-compartmental analysis was applied to estimate the area under the curve (AUC) 0–60 min. For the lowest inhaled dose (NHP#5, 0.5 μg/kg), all values fell below the lowest level of quantification.

### Quantification of ^11^C-VC-002 binding

Following preparatory checks, the total plasma ^11^C-VC-002 radioactivity concentration, i.e., without metabolite correction, was selected as the input function for quantification since metabolites were assumed to also enter the lung tissue. The multi-linear variant of the Logan graphical analysis method was used to quantify radioligand binding [12, 13]. The method provides an estimate of the total volume of distribution (*V*_*T*_), which serves as an index of total radioligand binding in the tissue. *V*_*T*_ is a common outcome parameter used in neuroreceptor quantification that is an estimate of total binding, corresponding to the ratio of radioactivity concentration in tissue over that in plasma at equilibrium (mL/cm^3^) [14]. Compared to traditional compartmental models, the advantage of the Logan quantification is that it provides a robust estimate of total binding irrespective of the compartmental configuration as long as there is a component of reversible specific binding.

The quantification was carried out at a voxel level using wavelet-aided parametric imaging (WAPI) [13, 15]. The implemented WAPI method provided voxel-wise parametric images of *V*_*T*_ and its standard error (SE) [16]. To obtain a single *V*_*T*_ (and SE) estimate for the target organs, the voxel-wise estimates were pooled using a weighted average where the weight for each voxel was proportional to the inverse voxel-wise variance (squared SE) in an analogous way as it has been described for a random effects meta-analysis [17].

Additional details can be found in Additional file 1.

## Results

Five non-human primates were enrolled in the study based on availability and examined according to the protocol. No adverse events were observed during or after the 18 PET-measurements performed in the study.

### Ipratropium pharmacokinetics after inhalation and infusion

**Fig. 1:**
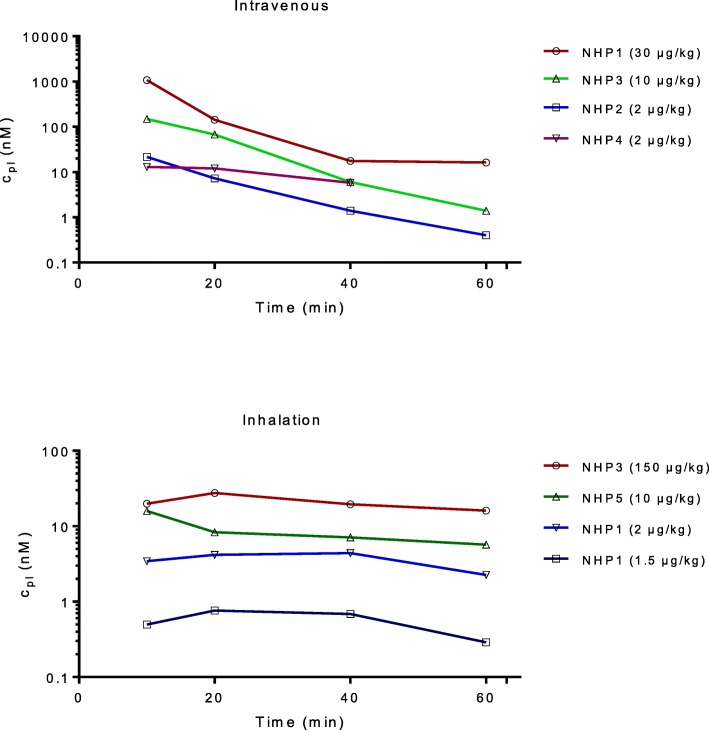
Plasma ipratropium concentration during time of PET data acquisition following administration via intravenous infusion and inhalation. Note the different log scales on the *Y*-axes

The concentration of ipratropium in the plasma was determined prior to and under each post-dosing PET measurement. Following intravenous infusion of ipratropium (2–30 μg/kg), the concentration in the plasma was initially high where after it rapidly decreased (Fig. 1). After inhalation of a nebulized solution of ipratropium (0.5–150 μg/kg, metered dose), the drug concentration in plasma was more than an order of magnitude lower than that observed after infusion. For the lowest inhaled dose (NHP5, 0.5 μg/kg), the plasma ipratropium concentration was below the limit of detection.

### Descriptive analysis of ^11^C-VC-002 disposition

**Fig. 2:**
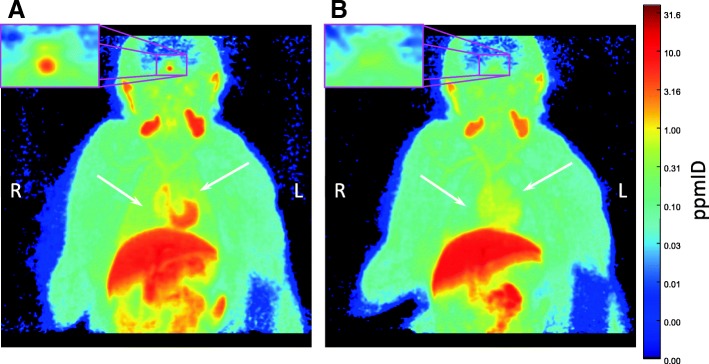
Coronal slices of summation PET images (3–60 min) showing the distribution of radioactivity after intravenous injection of ^11^C-VC-002 (in units of parts-per-million of the injected radioactivity dose, i.e., ppmID) in a non-human primate (NHP#3) at **a** baseline and **b** after inhalation of ipratropium (150 μg/kg). The highest inhaled dose was represented to provide a visual impression of the dosing effect. Arrows denote the lungs, and the insert shows the pituitary gland. The color scale is logarithmic

**Fig. 3:**
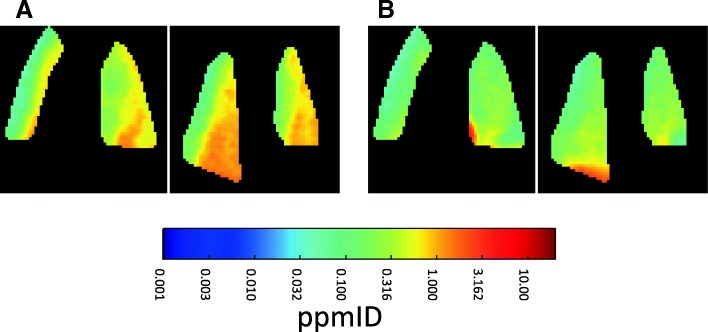
Coronal slices of summation PET images (3–60 min) showing the distribution of radioactivity after intravenous injection of ^11^C-VC-002 (in ppmID) in a non-human primate lung (NHP#1) at **a** baseline and **b** after intravenous administration of ipratropium (30 μg/kg). The highest intravenous dose was represented to provide a visual impression of the dosing effect. The color scale is logarithmic

The ipratropium-induced muscarinic receptor occupancy was investigated by pairwise PET measurements with ^11^C-VC-002 performed with the upper body in the field of view. In the baseline PET measurements, dense radioactivity was observed in several organs including the heart, kidneys, liver, lungs, pancreas, pituitary, and salivary glands (Fig. 2a, Fig. 3a). After post-dosing with ipratropium (Fig. 2b, Fig. 3b), regardless of the administration route, a reduction of radioactivity in the heart, lungs, pancreas, pituitary, and salivary glands was observed, whereas there was no evident effect on the radioactivity in the kidneys and liver.

**Fig. 4:**
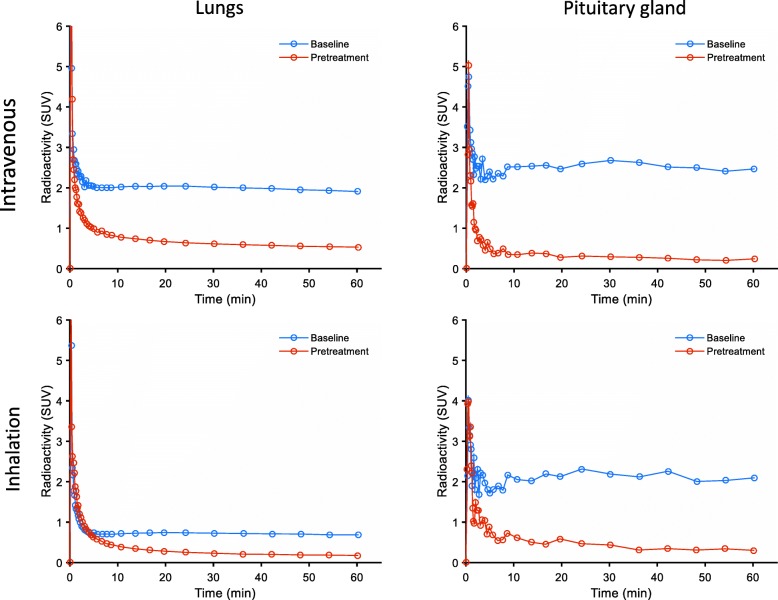
Example time-course for radioactivity in lungs and pituitary gland following intravenous injection of [^11^C]VC-002. The top TACs represent organ radioactivity at baseline and after post-dosing via intravenous infusion of ipratropium (NHP#1, 30 μg/kg). The bottom TACs represent organ radioactivity at baseline and after post-dosing via inhalation of ipratropium (NHP#3, 150 μg/kg)

For quantitative purposes, time-(radio) activity curves (TACs) were generated for the lungs and the pituitary gland. A rapid peak in the TACs for the lungs and the pituitary gland was observed both at baseline and post-dosing conditions (Fig. 4) where after radioactivity was at a plateau level from 10 min to the end of the PET-measurement. After post-dosing with ipratropium, regardless of the administration route, this plateau level was markedly reduced.

Following intravenous injection of the radioligand ^11^C-VC-002, radioactivity was measured in plasma and the fraction of radioactivity corresponding to unchanged radioligand was determined using radio-HPLC. The TACs for plasma were slightly higher at post-dosing compared to baseline conditions, regardless of administration route (Additional file 1: Figure S1). After drug treatment, the fraction corresponding to unchanged ^11^C-VC-002 appeared to be lower than at baseline conditions (Additional file 1: Figure S1, Additional file 1: Figure S2). The plasma protein binding of ^11^C-VC-002, determined by ultrafiltration, was 59% both after inhalation and intravenous drug treatment. Thus, no major differences were observed in plasma radioactivity, metabolism, or protein binding between intravenously treated animals and those treated via inhalation.

### Quantification of ^11^C-VC-002 binding

The Logan graphical analysis method, a standard method for quantification of radioligand binding [12], was used to quantify ^11^C-VC-002 binding at the voxel level. The total volume of distribution (*V*_*T*_) for the lungs and pituitary was determined for each PET measurement (see Tables 3 and 4). For both the lungs and pituitary, considerable intra- and inter-individual variability was observed in baseline *V*_*T*_ values. However, at post-dosing conditions, the *V*_*T*_ was reduced compared to that obtained at baseline, with one exception for the lungs at one of the lowest plasma ipratropium concentrations after inhalation administration (NHP #1 in Table 4). The ratio between *V*_*T*_ values obtained at post-dosing and baseline conditions (*V*_*T*_PT_/*V*_*T*_BL_) was then calculated and used as a surrogate for receptor occupancy.

**Table 3:** Total radioligand binding (*V*_*T*_ ± standard error) in the lungs and pituitary glands; ipratropium intravenous infusion dose and plasma exposure

NHP	Lungs		Pituitary gland		Ipratropium post-dosing	
	Baseline *V*_*T*_ (ml/cm^3^)	Post-dosing *V*_*T*_ (ml/cm^3^)	Baseline *V*_*T*_ (ml/cm^3^)	Post-dosing *V*_*T*_ (ml/cm^3^)	Dose (μg/kg)	Exposure, *C*_*pl*_ (nM)
#1	2.29 ± 0.019	0.35 ± 0.003	4.09 ± 0.272	0.20 ± 0.039	30.0	221.59
#2	0.39 ± 0.002	0.29 ± 0.001	1.48 ± 0.111	0.45 ± 0.046	2.0	5.93
#3	1.33 ± 0.011	0.30 ± 0.006	4.26 ± 0.346	0.42 ± 0.070	10.0	43.65
#4	0.66 ± 0.005	0.44 ± 0.003	5.93 ± 0.626	1.14 ± 0.131	2.0	7.09

**Table 4:** Total radioligand binding (*V*_*T*_ ± standard error) in lungs and pituitary gland; ipratropium inhalation dose and average plasma exposure

NHP	Lungs		Pituitary gland		Ipratropium post-dosing	
	Baseline *V*_*T*_ (ml/cm^3^)	Post-dosing *V*_*T*_ (ml/cm^3^)	Baseline *V*_*T*_ (ml/cm^3^)	Post-dosing *V*_*T*_ (ml/cm^3^)	Dose (μg/kg)	Exposure, *C*_*pl*_ (nM)
#1	0.32 ± 0.007	0.43 ± 0.005	2.08 ± 0.195	1.79 ± 0.166	1.5	0.55
#1	0.95 ± 0.012	0.29 ± 0.002	2.63 ± 0.202	0.42 ± 0.041	2.0	3.46
#3	0.52 ± 0.004	0.13 ± 0.001	0.91 ± 0.174	0.20 ± 0.071	150.0	19.36
#5	0.81 ± 0.010	0.59 ± 0.009	0.82 ± 0.251	0.63 ± 0.230	0.5	(0.25)^a^
#5	0.68 ± 0.013	0.11 ± 0.002	1.42 ± 0.348	0.17 ± 0.218	10.0	8.06

^a^Plasma exposure could not be measured, lower limit-of-quantification value used for model fitting

**Fig. 5:**
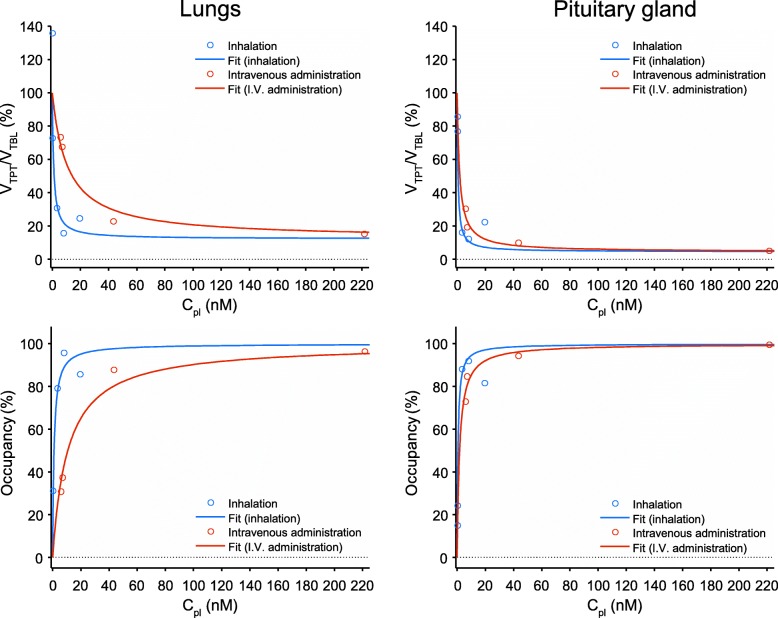
The relationship between plasma ipratropium concentration and changes in organ-specific binding of [^11^C]VC-002. Upper panels show changes in total binding (estimated using post-dosing over baseline *V*_*T*_ ratios) for the lungs and the pituitary gland, respectively. Lower charts show estimated changes in specific binding (i.e., target occupancy) for the two organs, respectively. The charts indicate subject- and occasion-specific data as scatter points and the results of model fitting as continuous curves. In detail, the scatter points for total binding were used to fit a model providing administration route and organ-specific parameter estimates for drug affinity (*K*_*i*_), as well as organ-specific estimates of baseline-specific binding (BP_ND_). The *K*_*i*_ in the lungs at inhalation was 1.01 nM (95% CI 0.36–2.82 nM), and in case of intravenous administration, it was 10.72 nM (95% CI 5.40–21.26 nM). These *K*_*i*_ values were significantly different at the 5% level (*p* value < 10^−4^). The baseline BP_ND_ in the lungs was 7.2 (3.6–14.4). The *K*_*i*_ in the pituitary at inhalation was 0.58 nM (95% CI 0.31–1.09 nM), and in case of intravenous administration, it was 1.72 nM (95% CI 0.93–3.17 nM). These *K*_*i*_ values were not significantly different at the 5% level (*p* value = 0.24). The baseline BP_ND_ in the pituitary gland was 21.4 (9.2–49.6)

The ipratropium plasma concentration was plotted versus the corresponding *V*_*T_*PT_/*V*_*T*_BL_ ratios (Fig. 5). The ratios could be described using the proposed model for inhibition of receptor binding (see Eq. 1, supporting information), which yielded estimates of baseline radioligand binding potential (BP_ND_) and ipratropium receptor affinity (*K*_*i*_). In addition, the model parameters presented in Fig. 4 were used to estimate receptor occupancy at a given plasma concentration. After inhalation, the estimated muscarinic receptor occupancy ranged between 30 and 98% (*n* = 4) whereas it ranged between 30 and 95% (*n* = 5) after intravenous administration.

### Inhalation produces a higher plasma concentration-derived apparent affinity for ipratropium

Ipratropium affected tissue binding (and thus the *V*_*T*_ ratio) in a dose-dependent fashion. At similar plasma ipratropium concentrations, administration via inhalation produced larger reductions in the *V*_*T*_ ratio for the lungs than drug administration via intravenous infusion. According to the two-route model fit for lungs, the plasma-derived apparent affinity for inhaled ipratropium (*K*_*i*ih_ = 1.01 nM) was roughly ten-fold higher than that observed for intravenously administered ipratropium (*K*_*i*iv_ = 10.72 nM). The estimated *K*_*i*_ values in the two-route model were significantly different (*p* < 0.001) and the *F* test comparing the two-route model and the pooled model indicated that the two-route model provided a significantly better fit (*p* = 0.002).

For the pituitary gland, there was no obvious difference in the shape of the fitted response curves between the two administration routes. According to the two-route model fit for the pituitary gland, the *K*_*i*ih_ was 0.58 nM and the *K*_*i*iv_ was 1.72 nM, but with no statistically significant difference (*p* = 0.24).

## Discussion

This is the first study that demonstrates relationships between drug dose, route of administration and target occupancy of a bronchodilator drug. Consistent with the view that inhalation provides local enrichment of drug in lung tissue, a higher target occupancy was observed in the lungs after inhalation compared to after intravenous infusion of ipratropium, at equivalent plasma drug concentrations. Following a more detailed analysis, the plasma concentration-derived apparent affinity (*K*_*i*_) for ipratropium was an order of magnitude higher after inhalation. A similar analysis of the exposure-occupancy relationship for the pituitary gland, an organ known to also be rich in muscarinic receptors, revealed no significant difference in *K*_*i*_ estimates between the two conditions. These results not only concur with the established understanding that inhalation leads to a therapeutic advantage in terms of maximizing the local (occupancy-driven) therapeutic effect whilst minimizing the systemically driven side-effects, but also provides a uniquely explicit quantification of such, indicating a 10-fold improvement in therapeutic window.

There is a large unmet need for biomarkers in respiratory drug development beyond forced expiratory volume. By using the muscarinic receptor system as a model system, this study demonstrates the feasibility of using PET for examination of drug-target engagement in pulmonary tissue and highlights a potential to close the knowledge gap between drug pharmacokinetics and pharmacodynamics in early clinical development. The presented methodology thus shows promise for aiding future drug development programs targeting underlying mechanisms and cure of respiratory disease.

### Quantification of ^11^C-VC-002 binding—method development and limitations

The effect of ipratropium on radioligand binding was evaluated by pairwise PET measurements performed on the same experimental day, in which the main outcome parameter was a ratio between radioligand binding at post-dosing over baseline conditions (the *V*_*T*_ ratio). By this experimental design, some of the noise that was introduced in a systematic fashion was anticipated to cancel out. Importantly, this provided the basis for a few key simplifications that were central for the study delivery from a practical perspective. These simplifications will be discussed in the following paragraphs.

No motion correction was performed on the PET data, which make the lungs appear spatially smoothed according to their motion through the breathing cycle. This was justified by the fact that the animals were ventilated during the study and that the lung movement was under the same rigorous control at both baseline and post-dosing conditions. The smoothing effect was thus assumed to be similar during each PET experimental session and thus cancel out.

In contrast to the brain, which is a relatively homogenous tissue, the tissue content in the lungs represents only ~ 12% of the total volume with the rest comprised predominantly of air (~ 72%) and blood (~ 12%) [18]. Therefore, binding parameters calculated without correcting for the inhomogeneity will likely be underestimated compared to the true binding values. Importantly, inspection of transmission data and of the initial slopes of the early radioactivity peak in the lungs indicated that air and blood content, respectively, did not substantially change between baseline and post-dosing conditions, which speak to the validity of these assumptions.

The lungs are in close vicinity to other organs with high radioactivity, e.g., the liver. The challenge with emission noise from nearby high uptake organs was primarily addressed by reducing the lung ROI so that voxels in the vicinity of these organs, such as the liver and the heart, were excluded from it. This was confirmed by the lack of high radioactivity voxels in the summation PET image within the final ROI. The influence of motion and interference from neighboring high radioactivity organs was also addressed by using an inverse-variance-weighted pooling of voxel-wise lung *V*_*T*_ estimates. The validity of this weighting scheme was supported by preliminary assessments, indicating that voxels with low *V*_*T*_ at the edge of the lungs (i.e., with more motion) and voxels high *V*_*T*_ closer to high radioactivity organs or containing more blood, such as near the back, tended to have higher absolute SE (and thus variance) than typical in-lung voxels, which, in contrast, tended to have rather similar SE, irrespective of *V*_*T*_. Various region of interest-based quantification methods were also evaluated during preliminary analyses, but proved to be overall less robust than the presented voxel-based method, yielding whole lung *V*_*T*_ values with higher SE and higher cross-subject variation (data not shown), thus supporting the choice of the voxel-based methodology.

Radioligand metabolites in the target tissue usually complicate accurate quantification of PET data. Since neither the lungs nor the pituitary gland has physiological barriers to plasma, radiolabeled metabolites enjoy free access to the tissues. In the present study, the total plasma radioactivity concentration was used as an input for the quantification in the lungs and pituitary gland, i.e., no metabolite correction was applied. This approach was expected to provide reasonable estimates of overall total organ binding under the assumption that the parent compound and its radiolabeled metabolites have similar kinetics of non-specific binding and, furthermore, that the labeled metabolites either do not bind to the target or bind with a similar affinity as the parent compound. Furthermore, Visser et al. demonstrated that 88% of the radioactivity in rat lung tissue at 15-min post-injection was constituted by parent radioligand [8].

The results presented herein indicate that despite all abovementioned challenges, it was possible to arrive at quantitative estimates on ^11^C-VC-002 binding in the non-human primate lungs and pituitary gland. Though the inclusion of additional animals on each dose point would have been beneficial from a statistical perspective, these quantitative estimates in turn provided consistent data for establishing a relationship between muscarinic receptor occupancy and plasma ipratropium concentration. Besides *K*_*i*_ estimates, the fitted occupancy model also incorporated binding potential representing ^11^C-VC-002 binding to muscarinic receptors at baseline conditions (see Fig. 5 with BP_ND_ estimates in the legend). The estimated value of about 7 in the lungs is substantial when compared to typical CNS radioligands and agrees with the known high affinity of ^11^C-VC-002 to muscarinic receptors and the high density of these receptors in the lung tissue [19]. Though *V*_*T*_ estimates are probably underestimated due to the relatively low tissue density (high air content), the BP_ND_ estimates were expected to be relatively unaffected by this because *V*_*T*_ ratios are used to derive BP_ND_ (see Eq. 1).

### Drug delivery via inhalation

The efficiency of drug aerosol inhalation is governed by the interplay between drug formulation, drug delivery device, and the recipient lung of the animal or patient. Following inhalation, drug deposition takes place in both conducting airways and alveolar lung regions where the gas exchange occurs. Although it was difficult to determine the exact regional drug deposition in the current experimental setup, previous studies have shown that drug deposition in mechanically ventilated lungs is more central (airway) relative to that observed in consciously inhaling patients [20]. Whilst the advantage of inhalation for separating occupancy of the lung and parotid gland was clearly demonstrated at the total receptor population levels, it is thus feasible that more proximal sub-populations of lung receptors have higher occupancy than the distal populations. Although the identity of the airways, whose caliber modulation by antimuscarinics is critically responsible for the reduction of airway resistance in COPD patients, are not clearly defined, there is empirical evidence suggesting that antimuscarinic drug deposition in the airways, rather than alveoli, is responsible for drug effect [21]. Much to our disappointment, however, preliminary attempts at examining regional differences in drug-induced receptor occupancy were unfruitful in the current study, largely due to considerable inter-individual variability in regional ^11^C-VC-002 binding. In this context, it is worth mentioning that a PET study with the nebulized radiolabeled drug could increase our understanding of regional drug distribution in the NHP lung under the current conditions. Nevertheless, future PET studies in human subjects are expected to provide a greater opportunity to image the proximal lung and thus aid in the identification of potential differences in drug-induced receptor occupancy between the airways and alveoli.

### Translating and applying the findings to early clinical drug development

The current study was conducted in non-human primates, and not in humans, for radiation safety purposes. Importantly, PET is a translational imaging technique, and from several perspectives, a study in human subjects may be conducted with greater ease and with more precision than the present study in non-human primates. Moreover, a greater number of blood samples can be sampled during a PET study in a human subject, and hence, the resulting blood curve usually contains considerably less noise. Finally, due to the larger size of the human lung, there is a greater opportunity to evaluate potential gradients in drug-induced muscarinic receptor occupancy between central and peripheral lung regions.

## Conclusions

This study demonstrates the feasibility and value of establishing quantitative receptor imaging for targets within the lung, with utility for addressing the myriad of important yet typically un-answered questions that arise in inhaled drug development: (i) which inhaled dose yields sufficient local target occupancy to warrant proof-of-principle testing in larger clinical studies? (ii) what is the consequence and dose-dependency of systemic spill-over on peripheral organ occupancy? how effectively are the peripheral lung regions targeted versus larger airway tissue? and (iii) how does (a required change of) the inhaled formulation or device affect all of the above? These are all questions that can be potentially addressed by target occupancy quantification by PET in the pre-clinical and clinical setting, and it is envisioned that PET imaging will play a central role in the development of novel treatment paradigms for the respiratory disease pandemic.

## Additional file

Additional file 1: **Figure S1.** Time-course for radioactivity in plasma following intravenous injection of [^11^C]VC-002. Average radioactivity concentrations across all NHPs examined at baseline (*n* = 9) and after pre-treatment via intravenous infusion (*n* = 4) or inhalation (*n* = 5) of ipratropium. **Figure S2.** The fraction of radioactivity corresponding to parent radioligand in plasma following intravenous injection of [^11^C]VC-002. Average radioactivity concentrations across all NHPs examined at baseline (*n = 9*) and after pre-treatment via intravenous infusion (*n* = 4) or inhalation of ipratropium (*n* = 5). **Figure S3.** The relationship between receptor occupancy in the lungs and pituitary at given plasma concentrations. Note the shift towards higher occupancy in pituitary gland after intravenous administration. **Figure S5.** PET images from each experimental session. **Figure S6.** Time-course for radioactivity in venous and arterial blood following intravenous injection of [^11^C]VC-002. (DOCX 1861 kb)

## Abbreviations

ABSS: Automatic blood sampling system
AUC: Area under the curve
BP: Binding potential
*C*_pl_: Plasma concentration
iv: Intravenous
*K*_*i*_: Inhibitory constant
LC: Liquid chromatography
MS: Mass spectrometry
NHP: Non-human primate
PBS: Phosphate-buffered saline
PET: Positron emission tomography
SE: Standard error
TAC: Time-activity curve
*V*_*T*_: Total distribution volume
WAPI: Wavelet-aided parametric imaging
