# Supplementary material for: Pulmonary PET imaging confirms preferential lung target occupancy of an inhaled bronchodilator
Source: EJNMMI Res. 2019 Jan 29;9:9. doi: 10.1186/s13550-019-0479-8 (PMC6890867; doi:10.1186/s13550-019-0479-8)
Supplement: Supplementary file 1 — Figure S1. Time-course for radioactivity in plasma following intravenous injection of [11C]VC-002. Average radioactivity concentrations across all NHPs examined at baseline (n = 9) and after pre-treatment via intravenous infusion (n = 4) or inhalation (n = 5) of ipratropium. Figure S2. The fraction of radioactivity corresponding to parent radioligand in plasma following intravenous injection of [11C]VC-002. Average radioactivity concentrations across all NHPs examined at baseline (n = 9) and after pre-treatment via intravenous infusion (n = 4) or inhalation of ipratropium (n = 5). Figure S3. The relationship between receptor occupancy in the lungs and pituitary at given plasma concentrations. Note the shift towards higher occupancy in pituitary gland after intravenous administration. Figure S5. PET images from each experimental session. Figure S6. Time-course for radioactivity in venous and arterial blood following intravenous injection of [11C]VC-002. (DOCX 1861 kb) [file 13550_2019_479_MOESM1_ESM.docx]

Supplementary Materials

Pulmonary PET Imaging Confirms Preferential Lung Target Occupancy of an Inhaled Bronchodilator

**Authors:** Magnus Schou^1,2^*, Pär Ewing^3^, Zsolt Cselenyi^1^, Markus Fridén^3,4^, Akihiro Takano^2^, Christer Halldin^2^ and Lars Farde^1,2^

**Affiliations:**

^1^PET Science Centre, Precision Medicine and Genomics, IMED Biotech Unit, AstraZeneca, Karolinska Institutet, Sweden

^2^Department of Clinical Neuroscience, Center for Psychiatry Research, Karolinska Institutet and Stockholm Country Council, SE-171 76 Stockholm, Sweden

^3^Respiratory, Inflammation and Autoimmunity IMED Biotech Unit, AstraZeneca, Gothenburg, Sweden

^4^Translational PKPD, Department of Pharmaceutical Biosciences, Uppsala University, Uppsala, Sweden

*Author wo whom correspondence should be addressed.

Email: [magnus.schou@astrazeneca.com](mailto:magnus.schou@astrazeneca.com), tel: +46851775598

# **Materials and Methods**

## Drug administration via inhalation

Ipratropium bromide, or Atrovent®, was obtained as a 0.5 mg/mL solution that was diluted with saline to the desired concentration for the nebulization study. The Aeroneb Pro™ nebulization device (Dolema AB, Sweden) was used for the inhalation. In brief, Aeroneb Pro™ was connected to the respirator using a T-junction via a pediatric intubation tube with an internal diameter of 4 mm. A solution of ipratropium bromide (4 mL) was then nebulized into the endotracheal tube during 15 min.

## Arterial and venous blood sampling

Blood samples (1–2 mL) at 2.5, 10, 20, 40 and 60 min were used for determination of unchanged [^11^C]VC002 and radioactive metabolites in plasma. To compare with the results of arterial blood samples, venous blood samples were also drawn at 10, 20, 40 and 60 min for measurement of radioactivity in whole blood and plasma as well as for metabolite analysis

A blood sample was taken at approximately 5 min before radioligand injection to allow for determination of the free fraction in plasma (*2*).

Blood samples (0.5 mL) were also taken before drug treatment start and at -5, -1, 30 and 63 min after the radioligand injection to assess the ipratropium concentration in plasma during the infusion and inhalation PET measurements. Ipratropium was quantitated in arterial and venous plasma using LC-MS/MS. Non compartmental analysis was applied to estimate area under the curve (AUC) 0-60 minutes using Phoenix™ WinNonlin® 6.3.0 (Pharsight, Sunnyvale, CA).

Regions of interest (ROIs) were delineated for the lung and brain on the summation PET images. The ROIs were applied to the dynamic PET data and the time activity curves of each organ were generated.

## Determination of plasma input function

The fraction of plasma radioactivity corresponding to unchanged drug in plasma was determined as previously described for other PET radioligands (*3*). Briefly, the plasma samples were deproteinized with acetonitrile and analyzed by gradient high-performance liquid chromatography with radiodetection. The metabolite corrected arterial input function was generated by connecting the ABSS curve with the interpolated curve from the manual blood samples. Corrections for dispersion, plasma radioactivity concentration, and radioactive metabolites were applied to generate the time curve for parent radioligand in plasma according to previously described procedures (*2*).

## Quantification of [^11^C]VC-002 binding

## Estimation of occupancy in lungs and plasma-derived apparent target affinity

The average plasma ipratropium concentration, plasma exposure, during the PET examination was used as an anchor point for assessment of drug induced changes in target receptor occupancy in case of either intravenous infusion or inhalation.

The ratio of total binding at pretreatment and baseline (i.e. V_T_PT_/V_T_BL_) was used as the dependent variable in the analysis. In detail, the relationship between the ratio of the pretreatment and baseline estimates of V_T_ and the average plasma exposure of ipratropium during the PET examination ($C_{pl}$) was evaluated using the following equation:

$\frac{V_{T\_PT}}{V_{T\_BL}}=\frac{1+{BP}_{ND\_BL}\times(1-\frac{C_{pl}}{C_{pl}+Ki})}{1+{BP}_{ND\_BL}}$ Equation 1.

where BP_ND_BL_ is the baseline binding potential of [^11^C]VC-002 in the target organ (an index of specific binding), and Ki is the apparent affinity of the drug to target receptors in the organ. The receptor occupancy (RO) was in turn calculated from the plasma concentration and the apparent affinity by using equation 2.

$RO=\frac{C_{pl}}{C_{pl}+K_{i}}$ Equation 2.

All experimental data, including results from both inhalation and intravenous pretreatment occasions, were fitted by a single, two-route model which incorporated two Ki estimates, one for each route of administration (i.e. Ki_iv_ for intraveneous and Ki_ih_ for inhalation administration, respectively), and a single estimate of BP_ND_BL_. To ensure non-negativity, the parameters were fitted on the logarithmic scale using non-linear optimisation in MATLAB (The MathWorks, Inc., Natick, Massachusetts, United States).

Alternatively, the model was also fitted by a pooled data model which ignored the way of administration and thus incorporated only one coomon Ki estimate (i.e. Ki_pooled_). The fit was weighted based on the inverse squared SE (i.e. inverse variance) of the V_T_ ratios, which were calculated from the SE of the V_T_ estimates using the following formula for error propagation:

${SE}_{r}=\sqrt{{SE}_{PT}^{2}\times\frac{1}{V_{T\_BL}^{2}}+{SE}_{BL}^{2}\times\frac{V_{T\_PT}^{2}}{V_{T:BL}^{4}}}$ Equation 3.

where SE_r_ is the SE of the V_T_ ratio, and SE_BL_ and SE_PT_ are the SE of the baseline and pretreatment V_T_'s, respectively.


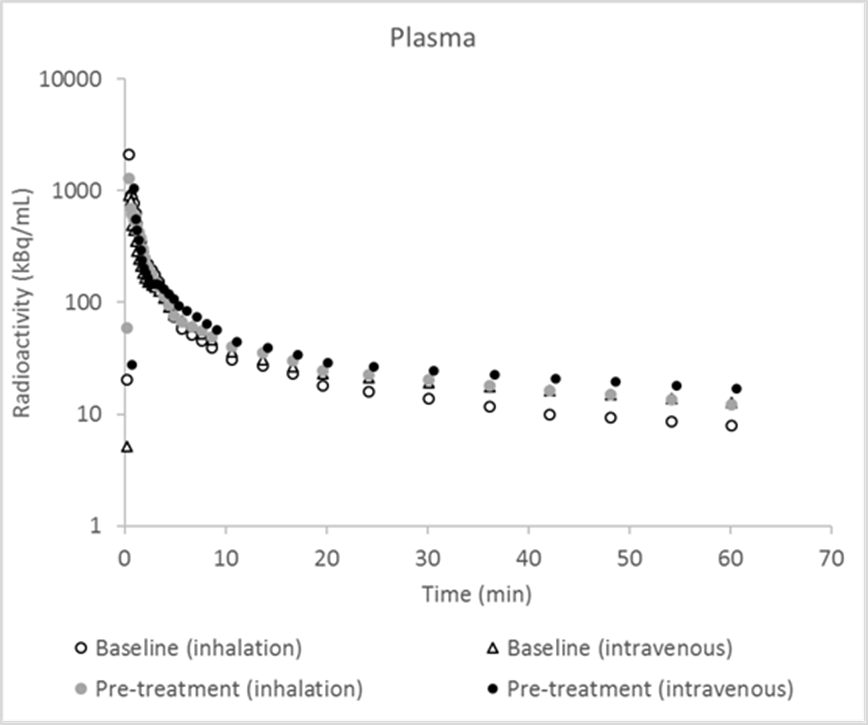


**Figure S1**. Time-course for radioactivity in plasma following intravenous injection of [^11^C]VC-002. Average radioactivity concentrations across all NHPs examined at baseline (*n*=9) and after pre-treatment via intravenous infusion (*n*=4) or inhalation (*n*=5) of ipratropium.


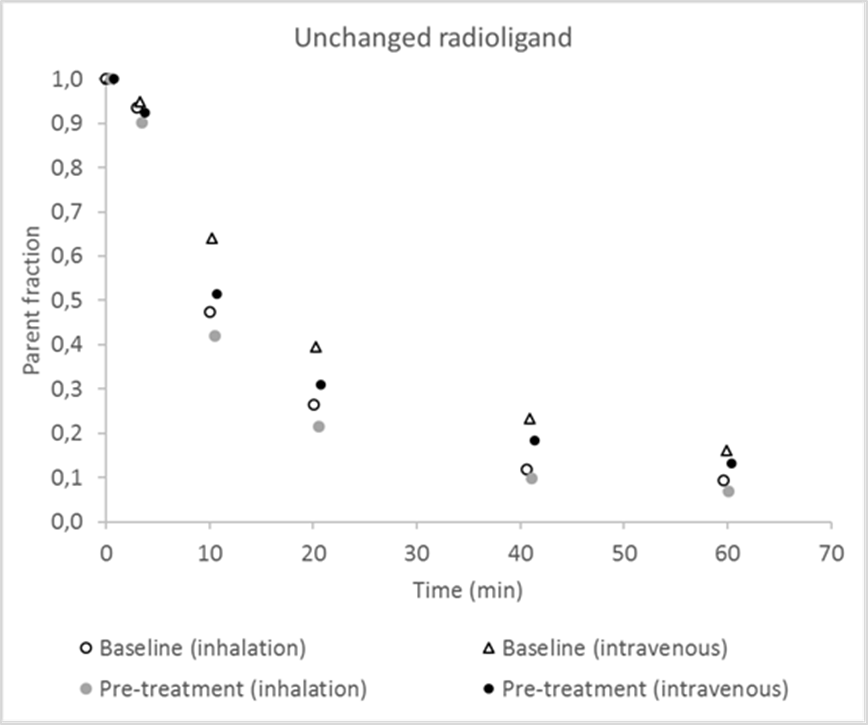


**Figure S2**. The fraction of radioactivity corresponding to parent radioligand in plasma following intravenous injection of [^11^C]VC-002. Average radioactivity concentrations across all NHPs examined at baseline (*n=9*) and after pre-treatment via intravenous infusion (*n*=4) or inhalation of ipratropium (*n*=5).

**Figure S3**. Relationship between receptor occupancy in lungs and pituitary at given plasma concentrations. Note the shift towards higher occupancy in pituitary gland after intravenous administration.


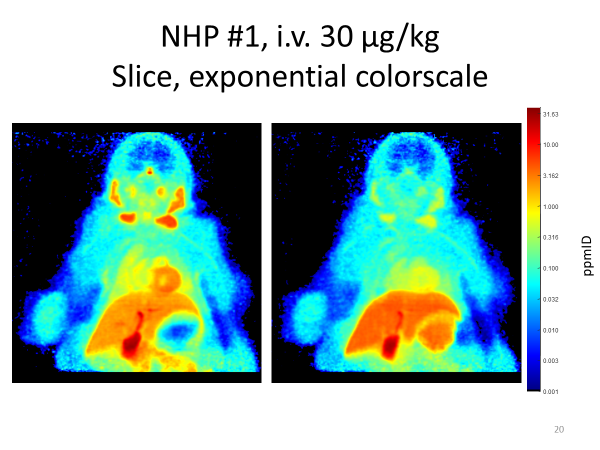


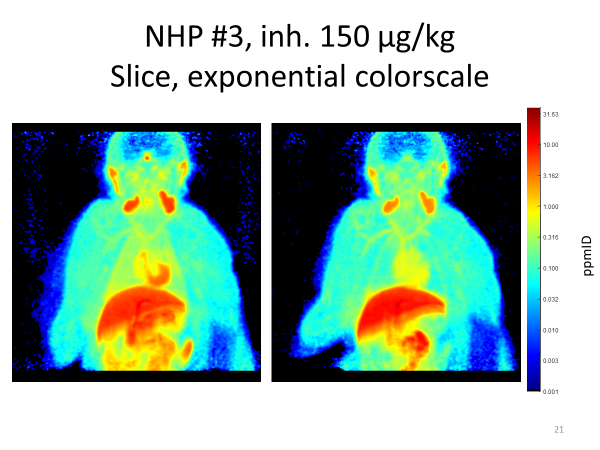


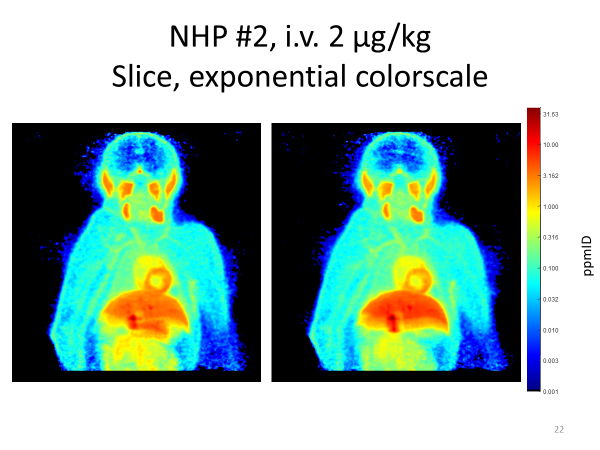


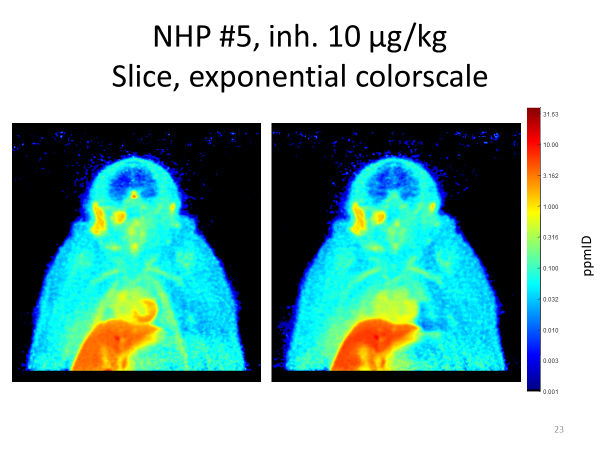


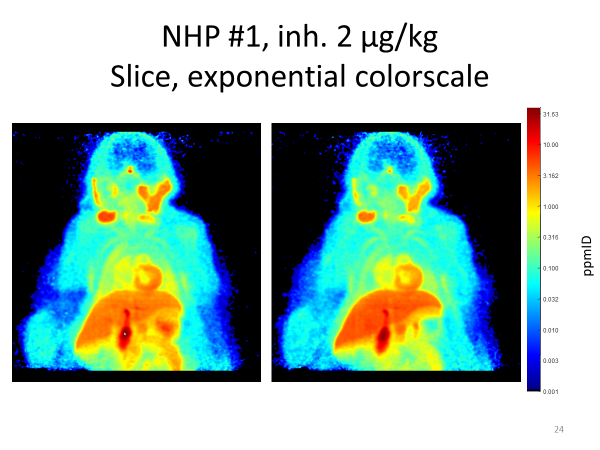

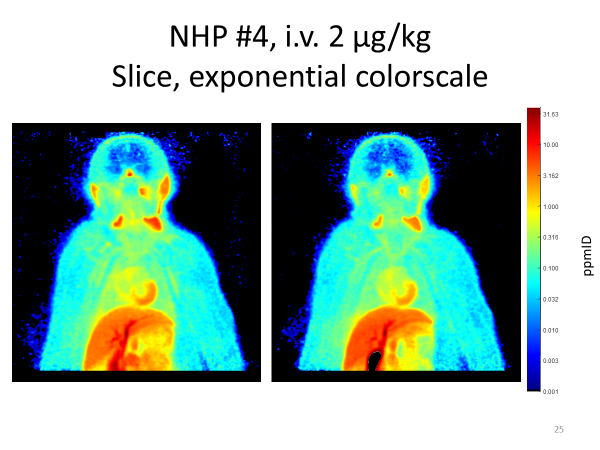


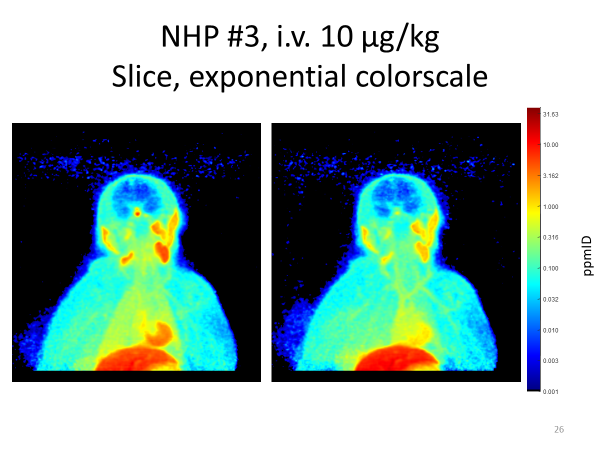


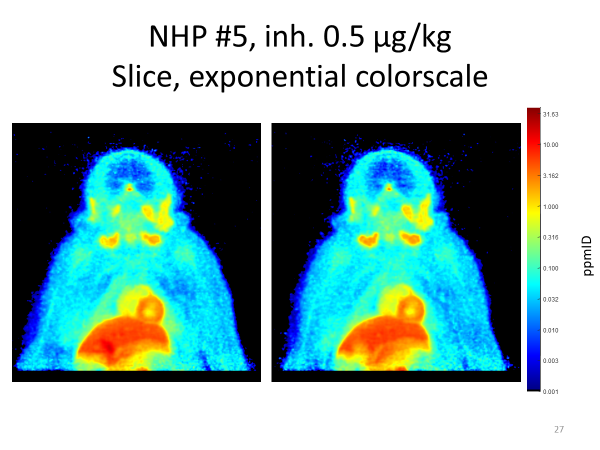


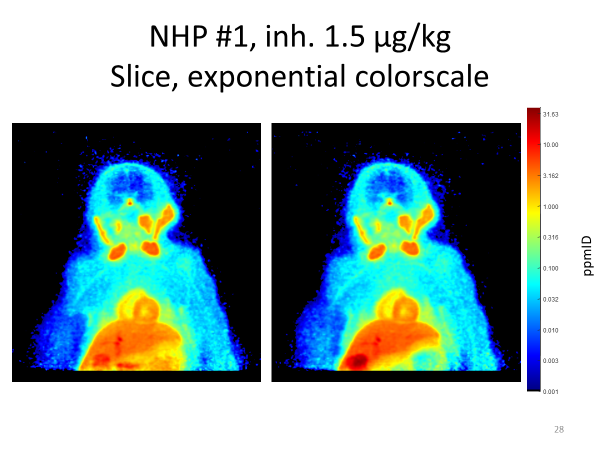


**Figure S5.** PET images from each experimental session.


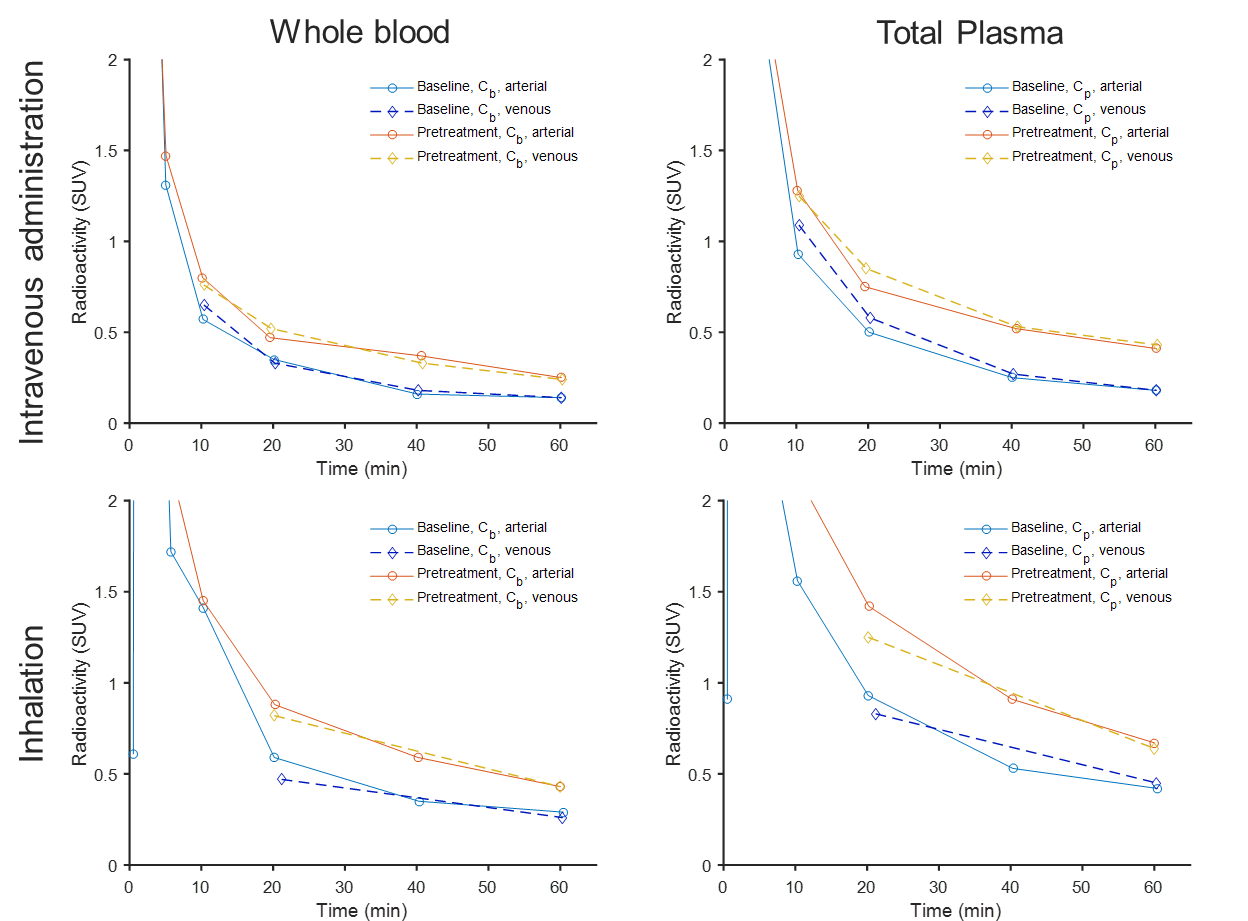


**Figure S6** Time-course for radioactivity in venous and arterial blood following intravenous injection of [^11^C]VC-002.

**References**

**1.** Varrone A, Sjoholm N, Eriksson L, Gulyas B, Halldin C, Farde L. Advancement in PET quantification using 3D-OP-OSEM point spread function reconstruction with the HRRT. *Eur J Nucl Med Mol Imaging.* 2009;36:1639-1650.

**2.** Schou M, Varnas K, Lundquist S, et al. Large Variation in Brain Exposure of Reference CNS Drugs: a PET Study in Nonhuman Primates. *Int J Neuropsychopharmacol.* 2015;18:pyv036.

**3.** Halldin C, Gulyas B, Farde L. PET studies with carbon-11 radioligands in neuropsychopharmacological drug development. *Current Pharmaceutical Design.* 2001;7:1907-1929.
